# Factor structure, reliability and criterion-related validity of the English version of the Problematic Series Watching Scale

**DOI:** 10.1192/bjo.2022.561

**Published:** 2022-08-24

**Authors:** Emanuele Fino, Mollie Humphries, Jake Robertson, Gábor Orosz, Mark D. Griffiths

**Affiliations:** NTU Psychology, Nottingham Trent University, UK; NTU Psychology, Nottingham Trent University, UK; NTU Psychology, Nottingham Trent University, UK; Sherpas Laboratory, Université d'Artois, France; NTU Psychology, Nottingham Trent University, UK

**Keywords:** Addiction components model, binge watching, factor structure, problematic series watching, online video streaming

## Abstract

**Background:**

Psychological research in the past decade has investigated the psychosocial implications of problematic use of on-demand online video streaming services, particularly series watching. Yet, a psychometric measure of problematic series watching in English is not available.

**Aims:**

The present study aimed to test the factor structure, reliability and criterion-related validity of the English version of the Problematic Series Watching Scale, a six-item self-report assessing problematic series watching, based on the biopsychosocial components model of addiction.

**Method:**

Participants were recruited from two UK university student samples. Study 1 (*n* = 333) comprised confirmatory factor analysis, reliability tests and item response theory analyses to test the original unidimensional model and investigate each item's levels of discrimination and information. Study 2 (*n* = 209) comprised correlation analyses to test the criterion-related validity of the scale.

**Results:**

There was a good fit of the theoretical model of the scale to the data (Comparative Fit Index = 0.998, Root Mean Square Error of Approximation = 0.024 [90% CI 0.000–0.093], Standardised Root Mean square Residual = 0.048), satisfactory reliability (*ω* = 0.79) and item levels of discrimination and information. The scale positively correlated with time spent watching series (*r_s_* = 0.26, *P* < 0.001) and negative affect (*r_s_* = 0.43, *P* < 0.001), and correlated negatively with positive affect (*r_s_* = −0.12, *P* > 0.05), mental well-being (*r_s_* = −0.25, *P* < 0.001) and sleep quality (*r_s_* = −0.14, *P* < 0.05).

**Conclusions:**

Results are discussed in relation to the ongoing debate on binge watching and series watching in the context of positive reinforcement versus problematic behaviour.

The recent proliferation of online video streaming platforms and services has resulted in global dramatic changes in consumer screen watching behaviour. Research has indicated distinctive situational and structural features facilitating their success and spread in several cultural contexts, including the unlimited access to shows, the affordable costs of subscriptions, being advertising-free, being available on multi-devices, and their wide offer of serialised shows.^1^ Other studies have highlighted the increasing personal autonomy underlying the offer as a major driver of their success.^2^

Research in the past decade has investigated the psychosocial implications of problematic use of online video streaming services, particularly ‘binge watching’ of television series.^3,4^ Although there is no consensus on its definition, the term 'binge watching' is broadly used to refer to a sustained usage of audio-visual content^2^ through digital media and in a serialised fashion.^3,5^ Some authors have questioned the extent to which binge watching represents a problematic behaviour, arguing that although excessive behaviours sometimes manifest as a repeated and prolonged engagement into a particular activity, they do not necessarily carry adverse biopsychosocial effects.^4^ Nevertheless, if comparing such behaviour to the more extensively studied phenomenon of problematic internet use, concerns arise as to whether some individuals might develop uncontrolled and dysfunctional patterns of binge watching, leading to mental and physical health consequences.^6^

The biopsychosocial components model of addiction^7,8^ posits that any behaviour can become addictive, and in that respect, six psychological components manifest in the affected individuals: salience, mood modification, tolerance, withdrawal, conflict and relapse. Salience indicates the prominence of the behaviour in an individual's mind. Mood modification represents the use of the behaviour to cope with stress, anxiety, depressive symptoms and with negative emotions generally. Tolerance refers to the progressively greater amount of time spent engaging in that behaviour to achieve the desired feeling (e.g., arousal or escape). Withdrawal represents the experience of cognitive distortion and negative feelings associated with a discontinuity in the behaviour, involving a series of psychological symptoms (from stress to insomnia, and even physical side-effects). Conflict indicates an individual's experience of struggling with the increasing concerns associated with the behaviour, to the point of interfering with their mental functioning, everyday activities and social relationships. Finally, relapse represents the tendency to revert to previous patterns of the behaviour as a consequence of a failed attempt to control or interrupt the dysfunctional cycle.

The biopsychosocial components model of addiction has been successfully adapted to the investigation of several online problematic behaviours, such as *YouTube* use,^5^ sexual behaviour and online pornography use,^9^ and most importantly, internet addiction.^10^ Recently, the addiction components model has also been used to operationalise the assessment of problematic series watching. A psychometric measure (i.e. Problematic Series Watching Scale; PSWS) was developed, tested and validated among Hungarian samples. However, to date, the English version provided by the authors has not been tested among an English-speaking sample, limiting its usability in other linguistic and cultural contexts.^11^

## Addictive versus problematic behaviours

Recent literature on problematic internet use has outlined an important theoretical distinction between problematic behaviours and addictive syndromes, with the former considered as being outside the range of pathological mental conditions rather representing ‘a distinct pattern of cognitions and behaviours’, often resulting in negative outcomes for daily life.^6^ In this sense, problematic behaviours have been located at the mid-point on a continuum of severity of implications for the individual versus addictive behaviours, the latter being intended to be at its extreme end.^6^ Nevertheless, problematic behaviours can negatively affect several aspects of an individual's life, such as their mental and physical health, educational and occupational performances, and social attachments,^6^ ultimately representing a risk factor for individual impairment.^6,12,13^ However, in light of the contemporary scientific debate on the risk for overpathologising everyday life, caution is required with interpreting excessive behaviours under the lens of problematic outcomes rather than a healthy and possibly positive reinforcement operated by the same behaviour.^3,4,14^

## Binge and problematic watching during the COVID-19 pandemic

Although binge watching and problematic video streaming use represent relatively understudied phenomena, some anecdotal observations and clinical case studies have recently been reported,^15^ concomitant with the global affirmation of models of subscription-based, on-demand streaming services. Some have highlighted that the phenomenon may be of even greater interest in the context of the COVID-19 pandemic and its associated mid- and long-term consequences, with social isolation, financial preoccupations and the relatively accessible and affordable services provided by on-demand platforms potentially increasing the risk for such activities to turn into a dysfunctional pattern of problematic behaviour.^16^ In particular, a study of 715 adults from the Italian population, conducted in the early stages of the COVID-19 pandemic and lockdown, showed that individuals spent, on average, more time watching series during that period compared with their pre-pandemic habits, and that was associated with anxiety and stress, especially among women. Interestingly, the authors found that both non-problematic and problematic TV series watching were associated with symptoms of anxiety and escapism, possibly acting as a psychological strategy to cope with the difficulties arising with the pandemic.^17^ Similarly, a study conducted among 1089 adults in the first half of 2021, showed that binge watching predicted stress, loneliness, insomnia, depression and anxiety, whereas time spent binge watching was found to increase the relevant symptoms.^18^ Another longitudinal study investigated series watching over a 6-week period during the first pandemic lockdown in Belgium, France and Switzerland. The authors found that male gender and social motives for series were associated with lower negative affect, whereas a loss of control of binge watching predicted negative affect over time.^19^

## Problematic series watching, internet addiction and mental well-being

Previous research has found internet addiction to be associated with negative affect, poor sleep quality, and impaired mental health and quality of life.^20–22^ In particular, a study indicated that approximately 27% of young Vietnamese individuals diagnosed with internet addiction reported sleep-related difficulties,^20^ whereas another study^21^ found that young individuals with internet addiction were more likely to report negative self-care, problematic daily routines, pain, discomfort, anxiety and depression. Similarly, a meta-analysis found internet addiction to be positively associated with alcohol abuse, hyperactivity, depression and anxiety.^22^

Interestingly, such results are consistent with recent literature on binge watching and problematic series watching. In particular, two self-report scales to assess series watching motives and binge watching engagement and symptoms have been recently developed,^3^ namely the Watching TV Series Motives Questionnaire (WTSMQ) and the Binge-Watching Engagement and Symptoms Questionnaire (BWESQ). The two scales resulted from exploratory and confirmatory analyses of motives for series watching and binge watching engagement and symptoms, respectively, in a sample comprised primarily of university students. The WTSMQ showed a four-factor model, including social motives, emotional enhancement, enrichment and coping/escapism, whereas the BWESQ showed a seven-factor model, including four ‘positive’ factors, namely engagement, positive emotions, desire savouring and pleasure preservation, as well as three factors representing dimensions of problematic behaviour: binge watching, dependency and loss of control.

They found significant correlations (*P* < 0.05) between the WTSMQ-Coping/escapism and positive affect (*r_s_* = −0.13), and between the former and negative affect (*r_s_* = 0.38), as assessed with the Positive and Negative Affect Schedule (PANAS).^23^ Notably, they also found a correlation of 0.39 between WTSMQ-Coping/escapism and scores on the Compulsive Internet Use Scale,^24^ which previous research found to correlate with depression and poor sleep quality.^25^ Similar patterns were observed for BWESQ-Binge watching (positive affect: *r_s_* = −0.07; negative affect: *r_s_* = 0.28; compulsive internet use: *r_s_* = 0.48), BWESQ-Dependency (positive affect: *r_s_* = −0.12; negative affect: *r_s_* = 0.31; compulsive internet use: *r_s_* = 0.46) and BWESQ-Loss of control (positive affect: *r_s_* = −0.14; negative affect: *r_s_* = 0.26; compulsive internet use: *r_s_* = 0.51). Other studies have reported positive correlations between problematic series watching and individuals’ self-development (*r* = 0.26), social interaction (*r* = 0.25), impulsive behaviour such as lack of perseverance (*r* = 0.24) and urgency (*r* = 0.25), harmonious series passion (*r* = 0.46) and obsessive series passion (*r* = 0.76),^26^ and between binge watching frequency and poor sleep quality (*r* = 0.15), cognitive pre-sleep arousal (*r* = 0.15) and somatic pre-sleep arousal (*r* = 0.09).^27^ Furthermore, a recent study reported 32% of poor sleepers as being binge viewers, similar to what was previously found in relation to internet addiction.^20,27^

## Development of an English version of the PSWS

Although the WTSMQ and the BWESQ have shown satisfactory measurement properties, they focus on series watching motives and binge watching; a specific measure of problematic series watching in English, to the best of our knowledge, does not currently exist. Having a reliable and valid measure of problematic series watching in English will carry important implications and potentially open the way to improve our current understanding of problematic series watching in several cultural contexts, with significant impact on the future agenda of research concerning problematic online behaviours.

In particular, as discussed in previous literature on other problematic behaviours, translating, adapting and validating psychometric measures in other cultural contexts carries a number of advantages. For example, Weatherly and colleagues note:

‘First, it would provide a single measure that was potentially useful to practitioners and researchers in multiple cultures. Second, such a measure could be used to identify differences at a cultural level … Third, if similar relationships are found between the contingencies maintaining … the behaviour and other measures of the same problematic behaviour in other linguistic and cultural contexts, then it could be argued that one of the important factors underlying … [such behaviour] had been identified’.^28^

Moreover, this will potentially enable researchers from several cultural contexts to address a number of unresolved theoretical and measurement-related questions about problematic series watching, such as (a) better differentiation between a ‘positive’ engagement from dysfunctional patterns of problematic watching among those presenting with an excessive and/or prolonged exposure to series watching; (b) clarification of the nomological network of problematic series watching, and (c) determination of the specific circumstances and factors prompting the activation, maintenance and reinforcement of excessive and problematic watching, in accordance with the recently proposed need for a behavioural analysis of problematic and addictive behaviours.^29^

## Research aims and hypotheses

For all of these reasons, in this research, we aimed to test the factor structure, reliability and criterion-related (concurrent) validity of the English version of the PSWS,^11^ using two UK samples of university students. In particular, Study 1 tested the original measurement model observed in Hungarian samples,^11^ the reliability of the model and the ability of the items to discriminate between individuals at different levels of the assumed latent dimension. Subsequently, Study 2 tested the criterion-related validity of the scale, hypothesising that PSWS scores would positively be associated with series watching time and negative affect, and negatively associated with positive affect, mental well-being and sleep quality.

## Method

### Study 1

#### Participants and procedure

Participants were UK undergraduate psychology students. The inclusion criteria were being aged at least 18 years, currently enrolled in an undergraduate psychology programme and having used any TV streaming service at least once in the past 12 months. A total of 405 students were contacted between January and June 2021, of which 358 completed the procedure and participated in Study 1, and 333 were retained for the analyses after preliminary data screening (see Results).

Participants were contacted and recruited online, mainly via the psychology department's institutional research participation scheme website, and via word of mouth and advertising on social media websites. They were told that this was a study on their use of online video streaming services and invited to complete an online survey through Qualtrics (Qualtrics, Provo, USA, https://www.qualtrics.com), an experience management platform. They were compensated for their time and effort with academic research credits, following the institution's recommendations. No financial incentives were offered. Electronic informed consent was obtained from all participants. Those who signed the consent form were initially screened for the inclusion criteria, and those who met the criteria were asked to complete the survey, at the end of which they were thanked and debriefed.

The authors assert that all procedures contributing to this work comply with the ethical standards of the relevant national and institutional committees on human experimentation and with the Helsinki Declaration of 1975, as revised in 2008. The School of Social Sciences Research Ethics Committee at Nottingham Trent University expressed favourable opinion on the ethics application (application number 2021/81 and amendment no. 2021/189).

#### Measures

The PSWS^11^ is a measure of problematic series watching, based on the addiction components model.^7^ Each item is scored on a five-point Likert scale from 1 (‘Never’) to 5 (‘Always’), asking participants to indicate how often during the past year they had thought, felt or behaved similarly to how it was described in each individual item. The scale includes six items, representing the components of salience, mood modification, tolerance, withdrawal, conflict and relapse, respectively. Originally, its psychometric properties were tested in two independent Hungarian samples, with the scale being internally consistent in both samples (Cronbach's α = 0.69 and 0.76). Total scores can be obtained by summing up individual item's scores.

Regarding the translation and adaptation procedure, the PSWS was developed on the basis of a seven-item measure of work addiction.^30^ A key difference between the two measures consisted in the replacement of the term ‘work’ with ‘series watching’. The PSWS items were originally translated from English to Hungarian, following the established and widely utilised protocol proposed by Beaton et al,^31^ for the cross-cultural translation and adaptation of psychometric measures, consisting of six stages (initial dual translation, synthesis of the two translations, blind back-translation, cross-cultural evaluation by a panel of experts, test of the pre-final version, and submission). The English version of the PSWS was finally provided by the authors in their published paper, and that was used in the present study.

#### Statistical analyses

Data were preliminarily screened for missing observations, unengaged responses (s.d. < 0.3) and multivariate outliers, the latter determined by estimating Cook's generalised distances from a factor-analytic model accounting for the six PSWS items loading onto the problematic watching latent dimension. The 0.5 quantile of the *F* distribution with *k* + 1 and *n* − *k* − 1 degrees of freedom (α = 0.001) was used as the cut-off value for detection. Two sets of analyses were performed on two different randomly extracted data-sets (*n_1_* = 166 and *n_2_* = 167) of the original sample (*N* = 333), respectively: Confirmatory Factor Analysis (CFA), and Item Response Theory (IRT) analyses.

As for CFA, the mean- and variance-adjusted weighted least-square estimation method was used to account for the ordinal nature of the data. To evaluate the fit of the model to the data, the study used the comparative fit index (≥0.95), root mean square error of approximation (<0.07) and standardised root mean square residual (<0.08). The reliability (internal consistency) of the PSWS was evaluated by means of the omega coefficient, considering values ≥0.7 as satisfactory. All the analyses were conducted through statistical programming language R version 4.0.4 for macOS, free software distributed under a GNU-style copyleft by R Core Team (R Foundation for Statistical Computing, Vienna, Austria, https://www.R-project.org/), and the following packages: lavaan,^32^ lordif,^33^ mirt^34^ and semTools: Useful Tools for Structural Equation Modeling (The Comprehensive R Network, https://cran.r-project.org/web/packages/semTools/index.html).

Regarding IRT, a unidimensional graded response model was fitted to the data, assuming the probability to endorse an item's response to be a function of the participant's location on the latent continuum of problematic series watching. The study estimated and evaluated items’ residual correlations from the model and tested the assumption of local independence. The probability density function was used on the latent continuum to estimate marginal reliability. Item's slopes (*α*), specifically their ability to discriminate between participants on the latent continuum, response category thresholds (*β*) and the item information function (IIF) were also estimated; the latter is considered as the degree of statistical information accounted for by the item. Item characteristic curves were used to inform the analysis and the evaluation of the model. Then, each item's invariance was examined by using logistic regression/IRT and the chi-squared likelihood ratio test (α = 0.001), specifically aiming to detect any possible differential item functioning across female and male participants.

### Study 2

#### Participants and procedure

Participants in Study 2 comprised 210 psychology students, recruited between June and October 2021, of which 209 were retained after data screening (one multivariate outlier was identified and removed). The same procedure, inclusion criteria and ethical considerations used in Study 1 were used in Study 2.

#### Additional measures

The 20-item PANAS^23^ comprises two ten-item subscales assessing positive affect (e.g. excitement, inspiration) and negative affect (e.g. feeling upset, afraid). In the original validation study, both scales were found to be reliable (Cronbach's α ranging from 0.86 to 0.90 for positive affect and 0.84 to 0.87 for negative affect; test–retest correlations ranging from 0.47 to 0.68 for positive affect and from 0.39 to 0.71 for negative affect). Total subscale scores were obtained by summing up individual item's scores.

The seven-item Short Warwick-Edinburgh Mental Wellbeing Scale (SWEMWBS)^35^ derives from the original 14-item Warwick-Edinburgh Mental Wellbeing Scale,^36^ with items representing mainly eudemonic well-being, rated on five response categories (1 = ‘None of the time’ to 5 = ‘All of the time’). Research showed that the scale is reliable (Cronbach's α = 0.85). Total SWEMWBS scores were obtained by first summing up individual item's scores, and then converting raw scores into metric scores.^36^

The Sleep Quality Scale (SQS)^37^ is a single-item measure of overall sleep quality. It requires respondents to think about the quality of their sleep overall, such as how many hours of sleep they usually get, how often they wake up during the night and earlier than they have to, and how refreshing they find their sleep. Respondents are then asked to rate their overall typical sleep quality on a Likert-type scale, with scores ranging from 0 to 10 (0 = ‘Terrible’, 10 = ‘Excellent’).

Finally, we asked participants to self-report how many hours they spend watching series, on average, per week.

#### Statistical analyses

The criterion-related (concurrent) validity of the PSWS was assessed with the second sample, after screening the data by using the same procedure used in Study 1. We used Spearman's correlations between the PSWS total scores and the other variables in the study.

## Results

### Study 1

Among the 358 students from the first sample who completed the study procedure, 285 (79.61%) self-reported as female, 67 (18.72%) self-reported as male, four (1.12%) self-reported as non-binary gendered and two (0.56%) preferred not to report their gender. Their minimum and maximum self-reported ages were 18 and 49 years (mean 21.82, s.d. 5.00), respectively. Five participants showed missing observations, 19 displayed patterns of unengaged responses (s.d. < 0.3) and one participant was identified as a multivariate outlier. These responses were removed from the analytical data-set, leading to a final sample of 333 useful observations, later randomly split into two independent and approximately equally sized data-sets (*n_1_* = 166 and *n_2_* = 167). Tables 1 and 2 report PSWS items’ descriptive statistics and the Spearman's rho items’ intercorrelation matrix, respectively.

**Table 1:** Descriptive statistics (Study 1; *n* = 333)

Items (During the last year, how often have you … )	Mean	s.d.	Median	Minimum	Maximum	Skewness	Kurtosis
1. … thought of how you could free up more time to watch series?	2.36	1.01	2	1	5	0.27	−0.75
2. … spent much more time watching series than initially intended?	2.96	1.19	3	1	5	−0.14	−0.92
3. … watched series in order to reduce feelings of guilt, anxiety, helplessness and depression?	3.32	0.99	3	1	5	−0.50	0.02
4. … been told by others to cut down on watching series without listening to them?	1.59	0.9	1	1	5	1.60	2.1
5. … become restless or troubled if you have been prohibited from watching series?	1.84	1.03	1	1	5	1.00	0.09
6. … ignored your partner, family members, or friends because of series watching?	1.44	0.84	1	1	5	2.11	4.07

**Table 2:** Problematic Series Watching Scale, Spearman's rho correlation matrix (Study 1; *n* = 333)

	1.	2.	3.	4.	5.
Item 1					
Item 2	0.30***				
Item 3	0.41***	0.33***			
Item 4	0.42***	0.31***	0.25***		
Item 5	0.39***	0.28***	0.38***	0.47***	
Item 6	0.32***	0.28***	0.22***	0.54***	0.44***

***P < 0.001.

**Fig. 1:**
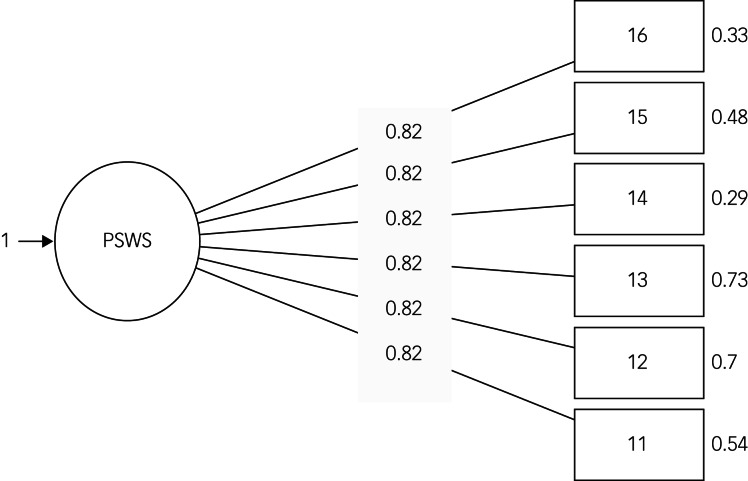
Confirmatory factor analysis (Study 1; *n*_1_ = 166). PSWS, Problematic Series Watching Scale.

Results from CFA (*n_1_* = 166) showed a satisfactory model fit (comparative fit index 0.998, root mean square error of approximation 0.024 (90% CI 0.000–0.093), standardised root mean square residual 0.048), confirming the fit of the model to the data. As shown in Figure 1, the items presented adequate standardised regression weights (0.51–0.84), with the solution being internally consistent (ω = 0.79).

**Fig. 2:**
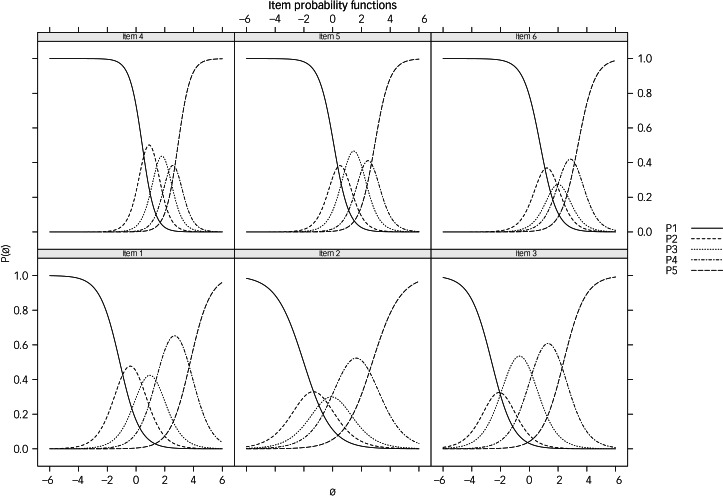
Item characteristic curves (Study 1; *n*_2_ = 167). P(θ), probability endorsing a category option; P1–P5, item response curves for category options 1–5.

An IRT graded response model was then fitted to the data (*n_2_* = 167). The model showed residual correlations higher than the estimated critical value (0.21) for all of the items, indicating possible local dependence. Satisfactory levels of marginal reliability were found (*r_xx_* = 0.76). Item characteristic curves showed patterns of ordered response categories for all the items, although items 2, 3 and 6 showed some overlaps of two categories (‘Rarely’, ‘Sometimes’), suggesting the utility of collapsing those categories (Fig. 2).

Item 4 showed the highest values of discrimination and information (*α* = 2.30, s.e. = 0.49, IIF = 1.60), followed by Item 5 (*α* = 1.90, s.e. = 0.37, IIF = 0.63), whereas Item 3 (*α* = 1.31, s.e. = 0.25, IIF = −0.41) and Item 2 (*α* = 1.03, s.e. = 0.22, IIF = −1.33) were the least informative and discriminative items. Table 3 reports detailed parameters, s.e. and information function for all the items.

**Table 3:** Graded response model, item discrimination parameters, category thresholds and information function (Study 1; *n_2_* = 167)

Items	*α*	s.e. *α*	*β*1	s.e. *β*1	*β*2	s.e. *β*2	*β*3	s.e. *β*3	*β*4	s.e. *β*4	IIF
Item 1	1.43	0.27	1.62	0.27	−0.46	0.22	−2.28	0.31	−5.39	0.81	−0.26
Item 2	1.03	0.22	2.08	0.27	0.71	0.2	−0.52	0.19	−2.85	0.34	−1.33
Item 3	1.31	0.25	3.44	0.42	2.09	0.29	−0.3	0.21	−3.12	0.38	−0.41
Item 4	2.3	0.49	−1	0.32	−3.2	0.53	−5.08	0.79	−6.7	1.18	1.6
Item 5	1.9	0.37	−0.16	0.25	−1.78	0.32	−3.8	0.51	−5.55	0.82	0.63
Item 6	1.71	0.36	−1.31	0.29	−2.85	0.42	−3.98	0.54	−5.77	0.9	−0.24

IIF, item information function, standardised values.

Items’ differential functioning was tested across female and male participants (*n_2_* = 165). The results from the likelihood ratio tests were not significant for any of the six items (α = 0.001), suggesting no differential item functioning.

### Study 2

In the second sample, consisting of 210 original observations, one multivariate outlier and no unengaged responses were found across all the measures. Therefore, the remaining 209 observations were used in the analyses. Among the 210 students, 166 (79.81%) self-reported as female, 39 (18.75%) self-reported as male, three self-reported as non-binary (1.44%) and two preferred not to report (0.96%). Their minimum and maximum self-reported ages were 18 and 29 years (mean 20.16, s.d. 1.46), respectively.

Table 4 presents descriptive statistics on all the measures, and Table 5 shows the results from the validity analyses. The PSWS positively correlated with time spent watching series (*r_s_* = 0.26, *P* < 0.001) and with PANAS negative affect (*r_s_* = 0.43, *P* < 0.001), and correlated negatively with PANAS positive affect (*r_s_* = −0.12, *P* > 0.05), SWEMWBS mental well-being (*r_s_* = −0.25, *P* < 0.001) and SQS sleep quality (*r_s_* = −0.14, *P* < 0.05), confirming the relevant hypotheses.

**Table 4:** Descriptive statistics (Study 2; *n* = 209)

Measures and items	Mean	s.d.	Median	Minimum	Maximum	Skewness	Kurtosis
PSWS 1	2.35	1.04	2	1	5	0.53	−0.28
PSWS 2	3.54	0.97	4	1	5	−0.74	0.55
PSWS 3	3.13	1.13	3	1	5	−0.25	−0.76
PSWS 4	1.61	0.97	1	1	5	1.84	3.05
PSWS 5	1.72	1	1	1	5	1.38	1.21
PSWS 6	2.17	1.1	2	1	5	0.6	−0.56
PANAS 1	3.21	0.9	3	1	5	−0.1	−0.39
PANAS 2	2.63	1.09	3	1	5	0.14	−0.95
PANAS 3	3.2	1.01	3	1	5	−0.2	−0.55
PANAS 4	2.57	1.17	2	1	5	0.35	−1
PANAS 5	2.62	1.12	3	1	5	0.28	−0.65
PANAS 6	1.9	1.07	2	1	5	0.96	−0.13
PANAS 7	2.1	1.17	2	1	5	0.69	−0.71
PANAS 8	1.67	0.91	1	1	5	1.27	1
PANAS 9	2.99	0.99	3	1	5	0.08	−0.51
PANAS 10	2.56	1.21	2	1	5	0.26	−1.02
PANAS 11	2.9	1.11	3	1	5	0.11	−0.89
PANAS 12	2.64	1.07	3	1	5	0.24	−0.68
PANAS 13	1.79	1.02	1	1	5	1.14	0.41
PANAS 14	2.68	1.17	3	1	5	0.1	−1.05
PANAS 15	2.98	1.22	3	1	5	−0.06	−1.04
PANAS 16	2.88	1.12	3	1	5	−0.13	−0.99
PANAS 17	2.9	1.02	3	1	5	−0.05	−0.78
PANAS 18	2.27	1.12	2	1	5	0.49	−0.79
PANAS 19	2.89	1.23	3	1	5	0.12	−0.97
PANAS 20	2.03	1.15	2	1	5	0.88	−0.31
SWEMWBS 1	3.18	0.95	3	1	5	−0.19	−0.46
SWEMWBS 2	2.91	0.98	3	1	5	0.05	−0.63
SWEMWBS 3	3	0.97	3	1	5	−0.01	−0.5
SWEMWBS 4	3.02	0.95	3	1	5	−0.05	−0.75
SWEMWBS 5	3.11	0.94	3	1	5	0.03	−0.73
SWEMWBS 6	3.23	1.05	3	1	5	−0.12	−0.82
SWEMWBS 7	3.44	0.96	4	1	5	−0.3	−0.51
SQS	5.89	1.88	6	2	10	−0.3	−0.54

PSWS, Problematic Series Watching Scale; PANAS, Positive and Negative Affect Schedule; SWEMWBS, Short Warwick-Edinburgh Mental Wellbeing Scale; SQS, Sleep Quality Scale.

**Table 5:** Spearman's rho correlation matrix (Study 2; *n* = 209)

Measures	1.	2.	3.	4.	5.	6.
PSWS						
PANAS (positive)	−0.12					
PANAS (negative)	0.43***	−0.09				
SWEMBWS	−0.25***	0.63***	−0.46***			
Time spent watching series	0.26***	−0.03	−0.02	−0.08		
SQS	−0.14*	0.40***	−0.22***	0.46***	−0.08	

PSWS, Problematic Series Watching Scale; PANAS, Positive and Negative Affect Schedule; SWEMWBS, Short Warwick-Edinburgh Mental Wellbeing Scale; SQS, Sleep Quality Scale.

*P < 0.05, ***P < 0.001.

## Discussion

The present study tested the factor structure, reliability and criterion-related (concurrent) validity of the PSWS, originally validated in Hungarian samples,^11^ among two UK samples of university students, using the English version provided by the authors in their published paper. We tested the relevant measurement model; its reliability; the ability of the items to discriminate between individuals positioned at different levels of the assumed problematic series watching latent continuum (Study 1); and the correlation between the PSWS and measures of time spent watching series, positive affect, negative affect, mental well-being and sleep quality (Study 2).

The results from Study 1 showed that the original measurement model was a good fit to the data, and that the solution was internally consistent, with all the items loading adequately onto the latent dimension. The IRT analyses indicated that three items required the collapsing of two categories to improve the substantive validity of the scale. Finally, regarding the criterion-related validity of the PSWS, the findings from Study 2 showed that PSWS scores were positively associated with time spent watching series and negative affect, and negatively associated with positive affect, mental well-being and sleep quality, confirming the relevant hypotheses.

### Implications of the results

The findings of the present study are promising and of great interest, but are far from conclusive and require further investigation. In fact, although the results contributed to evidence on the one-dimensional structure and the reliability of the PSWS, there is a significant lack of evidence in contemporary literature with regards to the addictive and/or problematic nature of series watching. Consistently, it must be noted that the extent to which excessive and/or prolonged series watching behaviour might constitute a risk factor for an individual's cognitive, emotional and functional impairment has yet to be defined. In this regard, the arguments provided in recent literature^3^ on the risk for overpathologising everyday life must be considered of foremost interest and importance.

Nevertheless, based on findings from other preliminary studies highlighting the potential of binge watching of representing an addictive behaviour,^38^ recent clinical and anecdotal reports among individuals seeking help to address symptoms of problematic use of online streaming platforms,^15^ and in the light of the societal and technological transformations happening globally, research is recommended to continue investigating the phenomenon, treating the results from the present study with caution and in the context of the wider evidence available to date. Moreover, findings of the present study will help researchers to address a number of unresolved theoretical and measurement-related questions on problematic watching, particularly (a) the extent to which an excessive, prolonged or serialised use of online video streaming services might represent a ‘positive addiction’^13^ versus a continuum of problematic behaviours, determining a low to mild, or even potentially high degree of individual impairment; (b) the degree of distinctiveness of problematic watching in relation to the wider study of problematic internet use; (c) the circumstances and the factors determining the activation, maintenance and reinforcement of binge watching and excessive watching behaviours, following the lines proposed for a behavioural analysis of problematic and addictive behaviours;^29^ (d) the prevalence of the phenomenon in the population, and the groups at higher exposure and possibly risk; and (e) how researchers and practitioners can validly assess an individual's change in their interaction with a potentially problematic or addictive behaviour. Regarding the latter point, it has been argued that it took years for a reliable psychometric instrument to be developed to assess pathological gambling, following the classification of it in the DSM-III, carrying dramatic consequences for those affected by the condition.

### Critical evaluation of the validity of the PSWS

In the present study, a positive association was found between PSWS total scores and time spent watching series and the PANAS negative affect scale, and a negative association with the PANAS positive affect scale, mental well-being (assessed with the SWEMWBS) and sleep quality (assessed with the SQS), providing preliminary evidence regarding the ‘problematic’ nature of series watching as measured through the PSWS. This could be explained by looking at findings from research showing moderate correlations between coping/escapism as motives for binge watching and negative affect, and zero-to-low correlations between binge watching and positive affect.^3^

However, we recommend future research to attempt to replicate and further test such relationships, ideally by means of exploratory factor analyses, to determine the factor space of problematic series watching in relation to correlates of excessive series watching, and by testing the validity of the PSWS, particularly in relation to the available measures of binge watching motives and engagement.^3^ In fact, in the study by Flayelle and collaborators, the authors explored four ‘positive’ factors, such as engagement, positive emotions, desire savouring and pleasure preservation, as well as three factors seemingly representing dimensions of problematic behaviour, such as binge watching, dependency and loss of control.^39^ Nevertheless, when looking at the correlations between the WTSMQ scores and the seven BWESQ factor scores, they were all low to moderate, whereas positive and moderate correlations were found between binge watching, dependency and loss of control assessed with the BWESQ, negative affect assessed with the PANAS and compulsive internet use assessed with the Compulsive Internet Use Scale.^3^ In turn, compulsive internet use has also been associated with depressive symptoms and poor sleep quality,^25^ and negative correlations have been found between the former three ‘negative’ BWESQ subdimensions and positive affect, prompting further investigation on the dysfunctional and problematic nature of series watching.^39^

Regarding mental well-being, findings from a recent study indicated that time spent binge watching, measured in terms of number of episodes watched, correlated with individuals’ free time and played a key role in the effect of binge watching on mental well-being.^40^ As for sleep quality, a noteworthy result emerging from the present study is the significant negative correlation between PSWS scores and sleep quality, consistent with the results from a recent study^27^ that found binge viewing to be increasing in its prevalence and potentially representing ‘a threat to sleep’. The authors of that study argued that a high cognitive arousal may serve as the key mechanism determining such phenomenon, and they concluded that excessive viewing time and cognitive arousal before sleep should be considered by further research and as possible targets for effective intervention in binge viewers.

Nevertheless, to avoid any speculations beyond the scope of the present study, we recommend further research to investigate the validity of the PSWS compared with other existing measures of binge watching and problematic series watching and common symptoms of mental distress, to help better define the construct both theoretically and operationally, and help to determine the extent to which the PSWS might help to shed a light on this relatively new and understudied phenomenon.

### General limitations

Beyond the discussion on the construct and criterion validity of the measure, the study also has other notable limitations. First, it was based on university student samples from one study discipline (i.e. psychology), requiring caution in the generalisation of the results to other populations. Second, the sample size was modest (although adequate for the testing of a six-item scale), requiring further testing in larger samples. Third, the test–retest reliability of the PSWS was not investigated. Fourth, participants were all recruited during the COVID-19 pandemic, which might have significantly skewed responses because of the social restrictions in place in the UK at the time when the data were collected, and the associated change in everyday habits and behaviours.

### Limitations of the confirmatory factor analytic approach

As suggested in recent literature,^3^ one of the main risk of a confirmatory approach to factor analysis might be represented by a possible overpathologisation of everyday behaviours such as television series watching, and the possibility that applying such models without a specific analysis of the validity of the model under investigation might generate problems in the interpretation of the model itself. In this regard, some have even argued that the confirmatory approach might even be conceptually untenable, overlooking alternative valid conceptualisations and proposing suboptimal treatment options,^41–45^ and for these reasons, further research exploring the factor space of problematic series watching in relation to the phenomenological underpinnings of binge watching and excessive series watching (e.g. motives, engagement and symptoms, e.g. as measured through the WTSMQ and BWESQ) is warranted.

In conclusion, our results show that the English version of the PSWS is internally consistent and adaptable to samples of UK university students, providing preliminary evidence on its criterion-related (concurrent) validity. Future research will benefit from comparing PSWS scores and compulsive internet use by means of validated and established measures like the Compulsive Internet Use Scale,^24^ aiming to differentiate between these two constructs and provide a clearer understanding of problematic series watching by means of a targeted analysis of the discriminant validity of the scale, along with further investigation of problematic series watching as excessive versus problematic behaviour.

## Data Availability

The data that support the findings of this study are available from the corresponding author, E.F., upon reasonable request.
